# Electronic Structure of Isolated Graphene Nanoribbons in Solution Revealed by Two-Dimensional Electronic Spectroscopy

**DOI:** 10.1021/acs.nanolett.3c02665

**Published:** 2024-01-08

**Authors:** Tetsuhiko Nagahara, Franco V. A. Camargo, Fugui Xu, Lucia Ganzer, Mattia Russo, Pengfei Zhang, Antonio Perri, Gabriel de la Cruz Valbuena, Ismael A. Heisler, Cosimo D’Andrea, Dario Polli, Klaus Müllen, Xinliang Feng, Yiyong Mai, Giulio Cerullo

**Affiliations:** † Dipartimento di Fisica, Politecnico di Milano, Piazza L. da Vinci 32, 20133 Milano, Italy; ‡ Department of Chemistry and Materials Technology, Kyoto Institute of Technology, 606-8585 Kyoto, Japan; § IFN-CNR, Piazza L. da Vinci 32, 20133 Milano, Italy; ∥ School of Chemistry and Chemical Engineering, Frontiers Science Center for Transformative Molecules, Shanghai Jiao Tong University, 800 Dongchuan Rd, Shanghai 200240, China; ⊥ Departamento de Física, Universidade Federal do Paraná, Caixa Postal 19044, 81531-990 Curitiba, Paraná, Brazil; # Max Planck Institute for Polymer Research, Ackermannweg 10, 55128 Mainz, Germany; ∇ Department of Chemistry and Food Chemistry, Technische Universität Dresden, Mommsenstrasse 4, 01062 Dresden, Germany

**Keywords:** graphene nanoribbons, ultrafast spectroscopy, two-dimensional electronic spectroscopy, inhomogeneous broadening, vibronic coupling

## Abstract

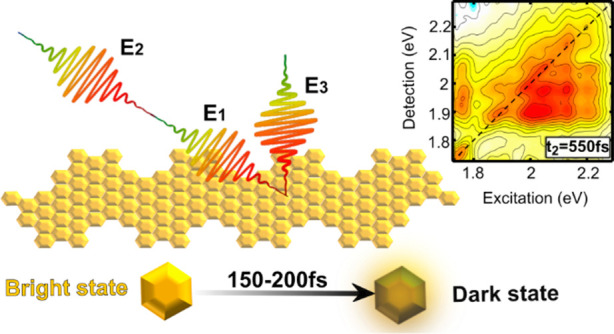

Structurally well-defined graphene nanoribbons (GNRs) are nanostructures with unique optoelectronic properties. In the liquid phase, strong aggregation typically hampers the assessment of their intrinsic properties. Recently we reported a novel type of GNRs, decorated with aliphatic side chains, yielding dispersions consisting mostly of isolated GNRs. Here we employ two-dimensional electronic spectroscopy to unravel the optical properties of isolated GNRs and disentangle the transitions underlying their broad and rather featureless absorption band. We observe that vibronic coupling, typically neglected in modeling, plays a dominant role in the optical properties of GNRs. Moreover, a strong environmental effect is revealed by a large inhomogeneous broadening of the electronic transitions. Finally, we also show that the photoexcited bright state decays, on the 150 fs time scale, to a dark state which is in thermal equilibrium with the bright state, that remains responsible for the emission on nanosecond time scales.

Graphene, a two-dimensional sheet of covalently bound carbon atoms, displays unique charge carrier physics^1^ and outstanding electronic, optical, and mechanical properties.^2^ Graphene is a gapless semimetal, with the valence and conduction bands consisting of two Dirac cones.^3^ It is possible to open a bandgap in graphene by lateral quantum confinement, in the form of one-dimensional nanostructures. One possibility is to wrap one layer of graphene on itself to form single-walled nanotubes (SWNTs);^4−6^ another is to slice it into narrow graphene nanoribbons (GNRs).^7−9^ Depending on their chirality, SWNTS can be either metallic or semiconducting, while GNRs have been shown to always have a nonzero bandgap.^8^ In both semiconducting SWNTs and GNRs, lateral quantum confinement gives rise to strongly bound excitonic transitions,^10,11^ tunable by varying their size and structure. This makes them promising candidates for applications to nanoelectronics,^12^ photonics,^13^ and quantum information.^14^

GNRs can be produced by either top-down or bottom-up approaches. Top-down methodologies^15−19^ cannot accurately control the width of the GNRs or the atomic structure of their edges. Bottom-up chemical synthesis, both surface-assisted^9,20,21^ and in solution,^22,23^ allows the production of uniform structures with atomically precise edges and 100% selectivity. Similarly, the electronic and optical properties of GNRs can be modeled by a top-down approach, whereby lateral boundary conditions are applied to a graphene sheet, or a bottom-up approach, which describes them as organic molecules of progressively larger size.^24^

Solution-phase synthesis of GNRs allows easy process scale-up, of great importance for applications, but results in strong aggregation effects due to π–π stacking interactions. Aggregation also hinders characterization of the linear and nonlinear optical properties of the GNRs,^25−29^ since it quenches the photoexcited states and prevents the study of the properties of the isolated nanostructures. Recently, we have demonstrated the synthesis of a new kind of GNR, decorated with pending Diels–Alder cycloadducts of anthracenyl units and *N*-*n*-hexadecyl maleimide (AHM).^30^ The AHM side groups have an ∼0.5 nm radius, which is larger than the interlayer spacing in graphite, effectively preventing π–π stacking of the GNRs.^31,32^ The AHM bulky groups and the long alkyl chains lead these so-called GNR-AHMs to show unprecedentedly high dispersibility in organic solvents and high photoluminescence (PL) quantum yields of ∼10%.^30^

GNR-AHMs display, above their ∼1.7 eV bandgap, a broad and mostly featureless absorption spectrum, for which the origin and the dynamics of the different electronic transitions have not yet been identified. Here we combine a variety of optical spectroscopy techniques, such as excitation emission maps (EEMs),^33^ ultrafast transient absorption (TA),^34^ and two-dimensional electronic spectroscopy (2DES),^35,36^ to study the nature and the dynamics of the excited states of isolated GNRs in solution. We find that the primary photoexcitations are strongly bound excitons, which are coupled to the characteristic vibrational modes of the GNRs and decay on an ultrafast (∼200 fs) time scale to a dark state in thermal equilibrium with the emitting state. 2DES reveals that the broad linear absorption spectra of GNR-AHMs can be well explained by a single excitonic transition with broad inhomogeneous broadening and strong vibronic coupling.

The GNR-AHM synthesis was described in our previous report (see the Supporting Information for details).^30^ The obtained GNR-AHM-1 and GNR-AHM-3 (following the same nomenclature as in ref (30)) consist, respectively, of 4 and 42 monomer units and possess a length (length dispersity) of 6(1.42) nm and 60(1.53) nm, respectively, according to the gel permeation chromatography results of their polyphenylene precursors before cyclodehydrogenation (see Table S1 in the Supporting Information). Figure 1a shows the chemical structure of a GNR-AHM, which consists of *n* repeat units of a monomer named C78 sandwiched between two end units, resulting in a chevron armchair structure with width between 1 and 1.7 nm. Figure 1b compares the linear absorption spectra of C78 to those of GNR-AHM-1 and GNR-AHM-3 in tetrahydrofuran. The monomer (black line) has its lowest energy transition at 2.16 eV, while the absorption spectra of the two GNRs (red and blue lines) are nearly identical, with the lowest allowed transition at ∼1.7 eV, red-shifted by 465 meV compared to the monomer. This implies that the electronic wave function spans a larger spatial domain in the GNRs when compared to the monomer, with excitonic delocalization resulting in a red-shifted absorption spectrum. However, given that GNR-AHM-1 and GNR-AHM-3 have similar absorption spectra, the delocalization is limited to a maximum of four monomeric units, in agreement with simulations on conjugated molecules.^37^ Although the lengths of GNR-AHM-1 and GNR-AHM-3 differ by 1 order of magnitude, the extent of π-delocalization is likely determined by the strong confinement along the width dimension, which alternates between 1 and 1.7 nm due to the chevron structure.

**Figure 1:**
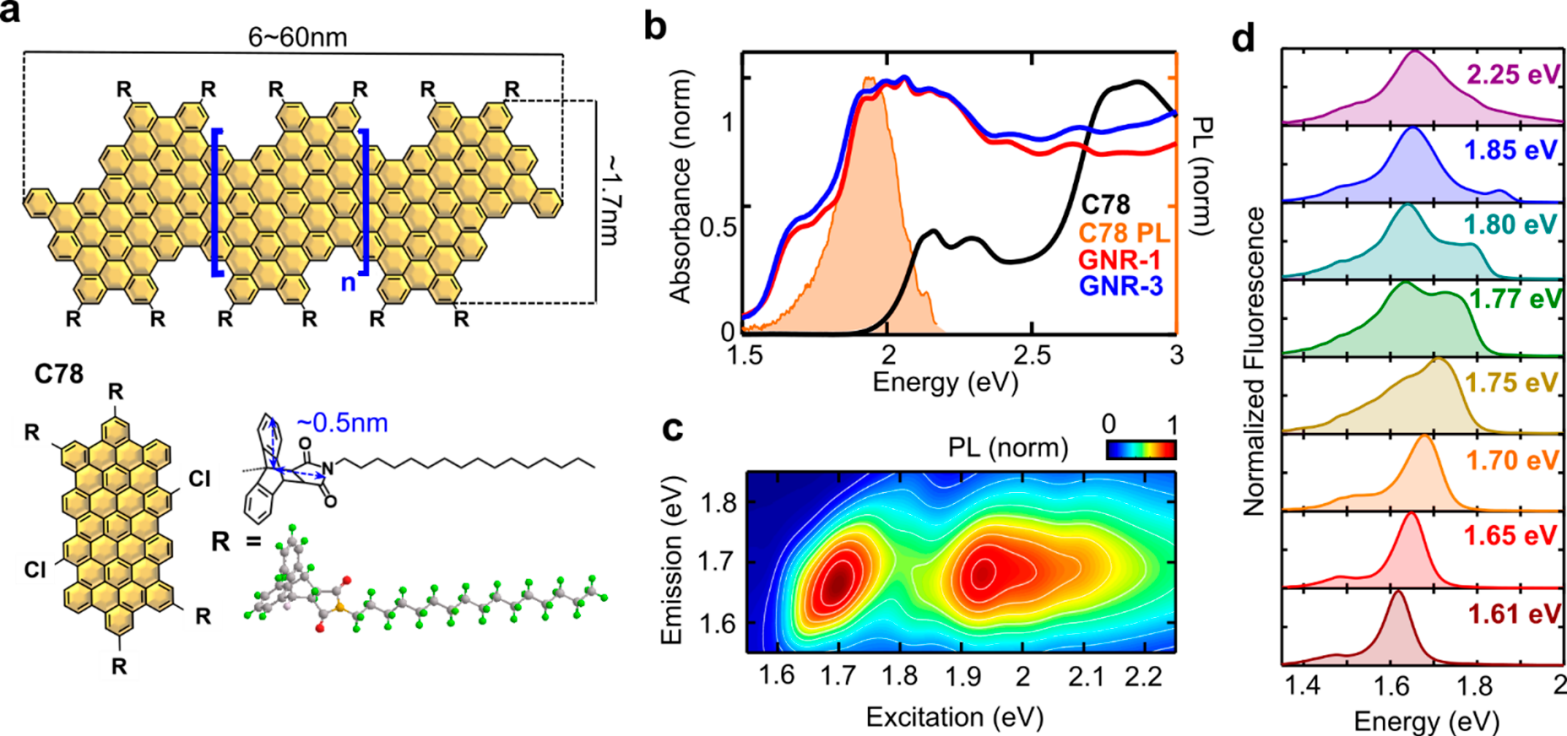
(a) Structures of GNR-AHM and its monomer, C78. (b) Linear absorption spectrum of GNR-AHM-1 (red), GNR-AHM-3 (blue), and C78 (black) and emission spectrum of C78 in shaded pink. (c) Steady-state excitation–emission map of GNR-AHM-3. (d) Vertical cuts of (c): PL spectra of GNR-AHM-3 as a function of excitation photon energy.

The bulky AHM side chains, while effectively hindering π–π interactions among different GNR backbones in the liquid phase, are also a source of steric hindrance. Previously, we observed that the GNR absorption close to the optical bandgap is strongly inhomogeneously broadened.^30^ Furthermore, no spectral diffusion on the characteristic solvent reorientation time scales was observed, with inhomogeneous broadening persisting beyond 1 ns.^30^Figure 1c reports a steady-state EEM of GNR-AHM-3, in which the PL spectrum is recorded as a function of excitation energy, enabling us to study the line shape broadening of GNRs on long time scales. The EEM was measured using a Fourier-transform approach,^38^ with a birefringent interferometer^39^ on the broadband excitation beam.

While C78 showed no excitation memory and a 200 meV Stokes shift (Figure S1a), the EEMs of GNR-AHM-3 and GNR-AHM-1 reveal a pronounced diagonal elongation for excitation energies in the range from 1.6 to 1.8 eV with an ∼20 meV Stokes shift. This is assigned to the distribution of conformers, each of which emits at its own transition energy. This pronounced excitation memory of the GNRs is in contrast with SWNTs, for which ultrafast spectral diffusion of excitons has been observed.^40^Figure 1d presents PL spectra for different excitation energies (vertical cuts of Figure 1c), where the emission peak shifts to higher energy by increasing excitation energy from 1.6 to 1.8 eV. For excitations above ∼1.75 eV, a second emission peak starts to appear at lower energies, creating a peculiar double peak structure of the PL spectrum. This can be assigned to the excitation of D and G vibronic replicas of the low energy conformers, similar to previous observations in SWNTs.^41^ For instance, 1.75 eV corresponds not only to the 0–0 vibronic transition of heavily strained conformers but also to the red-shifted 0–1 vibronic transitions of differently strained conformers. This peculiar effect is schematized in Figure S2.

We employ 2DES to identify the electronic transitions hidden in the heavily inhomogeneously broadened absorption spectrum of the GNRs. This technique is an extension of TA spectroscopy, which provides resolution in both excitation and detection energies. In 2DES the system is illuminated by three ultrashort laser pulses, two excitation pulses with delay *t*_1_ (the coherence time), and a detection pulse with delay *t*_2_ (the waiting time) with respect to the second pulse. The third-order nonlinear signal, emitted as a function of *t*_1_ and *t*_2_ and of detection time *t*_3_, is resolved in amplitude and phase by spectral interferometry. A Fourier transform with respect to *t*_1_ and *t*_3_ yields a correlation map between excitation and detection energies for each waiting time *t*_2_.^42^ A vertical cut of a 2DES map for given values of excitation energy and *t*_2_ yields the corresponding TA spectrum. 2DES simultaneously maximizes temporal and spectral resolution and enables detection of couplings and transfer processes between different electronic transitions. It was extensively applied to SWNTs,^43−46^ revealing energy transfer pathways between nanotubes of different chirality.

We perform 2DES in the partially collinear pump–probe geometry, employing two phase-locked collinear excitation pulses and one non-collinear detection pulse, whose differential transmission is measured by a spectrometer. In this geometry, the recorded maps correspond to absorption changes. The phase-locked excitation pulses are generated by a common-path birefringent interferometer.^47^ We use two broadband excitation/detection pulses (Figure S3): the first, with a near-infrared (NIR) spectrum spanning 1.24–1.8 eV, resonant with the band-edge absorption, and the second, with a visible spectrum spanning 1.73–2.4 eV, providing significant excess energy.

Figure 2a shows a 2DES map of GNR-AHM-3 in the NIR, following band-edge excitation, for *t*_2_ = 60 fs. The map is dominated by a strongly elongated positive peak along the diagonal, which is assigned to the combination of ground state bleaching (GSB) and stimulated emission (SE) of the inhomogeneously broadened excitonic transition. The map also shows a cross-peak for excitation at 1.8 eV and detection at 1.65 eV, which corresponds to SE to a vibronic replica of the ground state. The 2DES map at *t*_2_ = 1 ps (Figure 2a) does not evidence, apart from an amplitude reduction, any change in shape. The 49 meV antidiagonal width of the peak (Figure 2d) corresponds to the homogeneous line width of the GNRs and gives 84 fs dephasing time.^48^ The antidiagonal cut of the EEM, shown in Figure 2e, gives a very similar 47 meV line width, confirming the absence of spectral diffusion.

**Figure 2:**
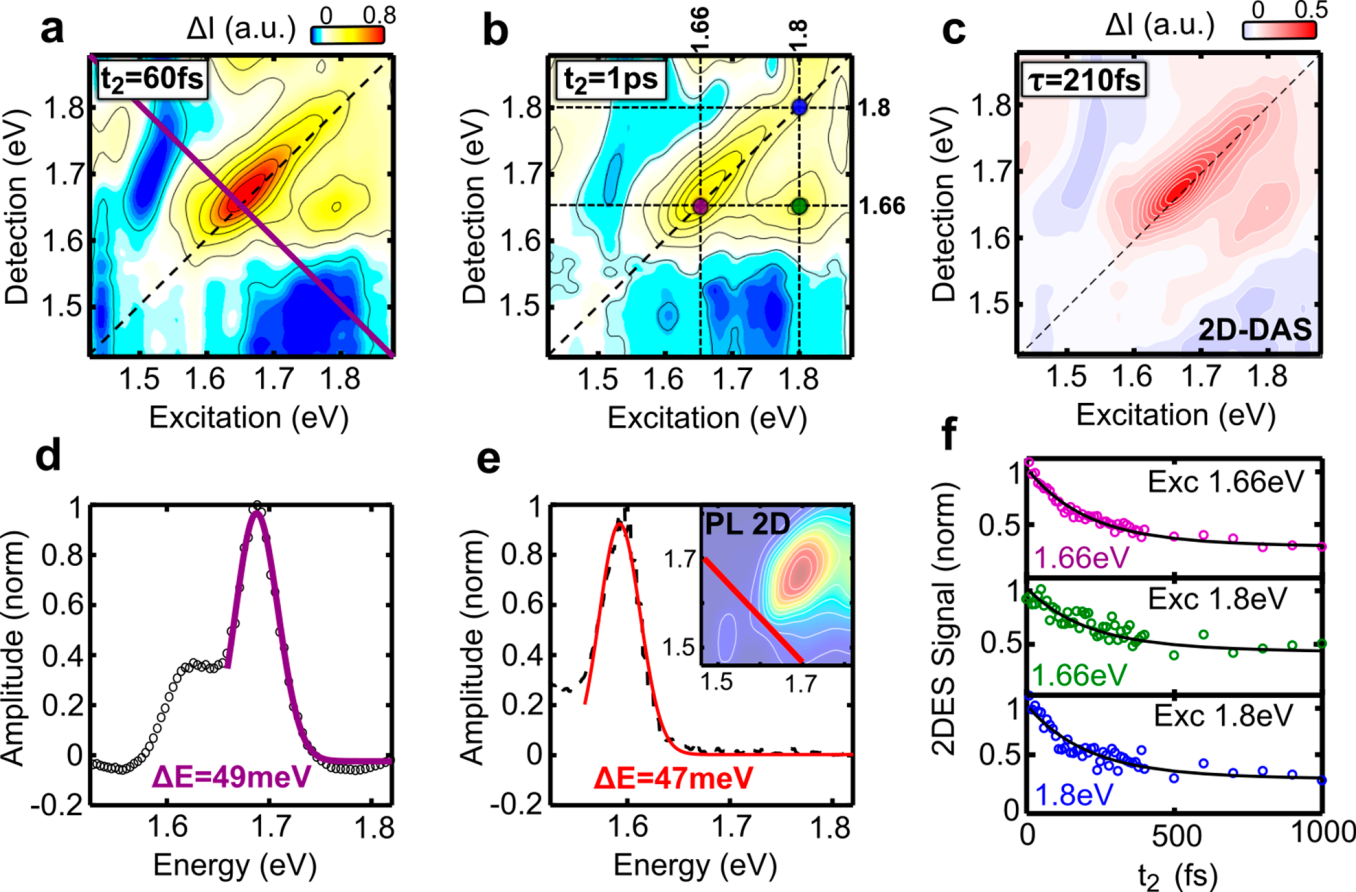
(a, b) 2DES maps of the GNRs at waiting times of (a) *t*_2_ = 60 fs and (b) *t*_2_ = 1 ps. (c) 2D-DAS map with the characteristic time 210 fs obtained by a global fit of the sequence of 2DES maps. (d, e) Antidiagonal cuts and Lorentzian fit of the main peak for the (d) 2DES data and (e) steady-state EEMp. (f) 2DES dynamics at the diagonal and cross-peaks marked in panel b.

To analyze self-consistently the dynamics of all 2DES spectral coordinates, we employ a global fitting routine in which *t*_2_-dependent traces for all pairs of excitation-detection energies are fitted simultaneously to a parallel decaying scheme consisting of a sum of exponentials.^49^ The fit provides the amplitude of each exponential component as a function of excitation and detection energies, called 2D decay associated spectra (2D-DAS). We satisfactorily fit the 2DES data set using only two exponential components. The shortest time constant is 210 fs (Figure 2c), whereas the second component has a fixed lifetime of nanoseconds, much longer than our 2 ps experimental time window.

Moving to excitation at higher energies (1.75–2.3 eV), Figure 3a shows an absorptive 2DES map at *t*_2_ = 550 fs using perpendicular excitation-detection polarizations, which minimizes broad photoinduced absorption signals that partly overlap with the GSB and SE in this spectral range.^30^ We observe a rich structure of diagonal and off-diagonal peaks, revealing that the broad absorption spectrum of the GNRs is due to the superposition of a distribution of coupled transitions.

**Figure 3:**
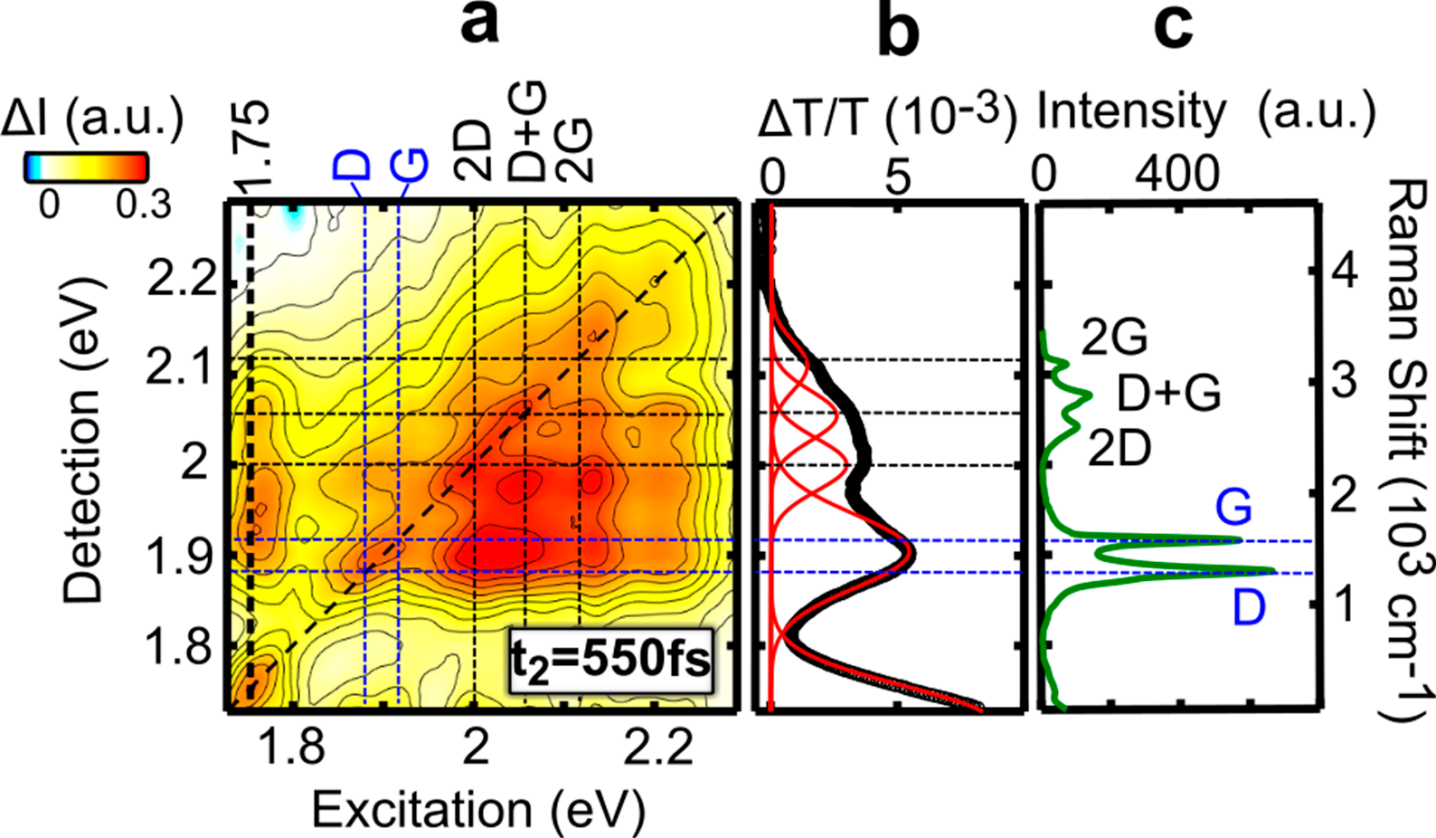
(a) 2DES map of GNR-AHM-3 at *t*_2_ = 550 fs with perpendicular pump and probe polarizations. (b) Vertical cut of the 2DES map at a 1.75 eV excitation energy and fit with a sequence of Gaussians. The respective peaks (eV) and widths (meV) are 2.110(39), 2.055(39), 2.000(41), and 1.904(56). (c) Steady-state Raman spectrum of GNR-AHM-3 excited at 532 nm.

We first test if vibronic coupling, known to be strong in carbon-based systems such as SWNTs^50,41^ and π-conjugated molecules,^51^ can explain the cross-peaks observed in our 2DES maps. We analyze a vertical cut of the 2DES map at a 1.75 eV excitation energy. Figure 3b shows that this spectrum can be fit to five Gaussian components. The energy separation between these components follows that expected from the Raman spectrum of the GNRs,^52^ reported in Figure 3c, with the first vibronic peak at ∼1.9 eV corresponding to a single band including the D and G Raman peaks and the broader high energy feature corresponding to the superposition of the 2D, D+G, and 2G peaks. This indicates that the rather featureless linear absorption spectrum of the GNRs consists of a single, inhomogeneously broadened transition with its vibronic replicas due to strong coupling to the D, G, 2D, D+G, and 2G Raman bands. In graphene the D mode is symmetry forbidden in Raman measurements and is only observed near the edges of graphene sheets or due to the presence of defects.^53^ Since the GNRs have a substantial number of edges, it is natural to expect a strong presence of the D mode in the Raman spectrum. These results are consistent with early time TA spectra acquired with narrowband excitation at 1.7 eV, shown in Figure S4, where two bands at 1.51 and 1.32 eV appear. These correspond to SE from the excited state to the ground state vibrational levels, and their energy spacing closely matches the measured Raman spectrum for GNR-AHM-3 (blue line in Figure S4).

While coherent oscillations are expected in the 2DES maps,^36^ an optimally designed experiment to extract them would be required to simultaneously cover the 1.4–2.4 eV spectral range while collecting complex-valued rephasing and non-rephasing signals,^54^ something our 2DES setup, optimized to measure population kinetics, cannot do. To detect oscillations, we employed broadband TA, which allows much higher signal-to-noise ratios. The data (Figure S6) reveals coherent oscillations corresponding to the D and G modes at 1310 and 1600 cm^–1^, in good agreement with the steady-state Raman spectra. We also observed low-frequency breathing modes near the lowest excitonic transition. Due to the dispersive nature of these modes with excitation energy and the strong inhomogeneous broadening of the samples, broadband TA is not an ideal technique to study them, but they are observed with different frequencies within the broad low frequency Raman band as expected.^52,55,56^

The strong coupling between electronic end vibrational degrees of freedom in this system, while not surprising when considering the GNR as a π-conjugated molecule, contrasts with the way optical properties of GNRs are usually modeled, neglecting vibrational degrees of freedom.^57,58^ Tries et al. modeled a GNR containing the same backbone as GNR-AHM-3^11^ and concluded that transitions between different electronic states accounted for the broad, multipeak structure of the excitonic absorption, obtaining a free carrier bandgap near 2.3 eV, in reasonable agreement with Liu et al., who studied a similar GNR in the solid state using scanning tunneling spectroscopy.^56^ Our data, on the other hand, can be fully explained by a single inhomogeneously broadened electronic transition coupled to the active Raman modes. While they cannot exclude the presence of other electronic transitions, they establish the critical role played by vibrational coupling and the local environment in the optical properties of GNRs. We note that this differs significantly from the case of SWNTs, where vibronic coupling, even if observed,^41^ does not dominate the optical response.^43−45^

Finally, we study the excited state dynamics in the visible region by recording 2DES maps for different values of *t*_2_ up to ∼2 ps. Panels a and b of Figure 4 compare 2DES maps for *t*_2_ = 60 fs and *t*_2_ = 1000 fs, obtained with parallel pump–probe polarizations, a scheme better suited to maximize the SE contributions. SE reflects the evolution of the excited state population and can be observed on and below the diagonal of the 2DES maps, with detection energy lower than the excitation energy. From 60 to 1000 fs the 2DES maps show a significant decrease in amplitude at and below the diagonal but no major changes in the positions and relative intensities of the peaks, as observed also in Figure 2a–c around the optical bandgap. This is confirmed by the 2D-DAS shown in Figure 4c, which indicates a decay with a 150 fs time constant at positions at and below the diagonal. Figure 4d presents kinetic traces overlaid with the fits at specific 2DES spectral coordinates, marked as green, blue, magenta, and orange dots in Figure 4b, revealing a substantial decay within 500 fs and a slower relaxation for longer times at coordinates on and below the diagonal. For coordinates above the diagonal (orange trace), the fast decay is not observed. These results are consistent with previous experiments^30^ which, with excitation covering the red side of the absorption spectrum (1.4–1.9 eV), revealed a similar fast decay of SE with an ∼250 fs time constant. It is interesting to note that, regardless of the vibronic peak that is excited, the relaxation of the excited state always follows the same fast 150 fs kinetics, as attested by the good quality of the fit in Figure 4d.

**Figure 4:**
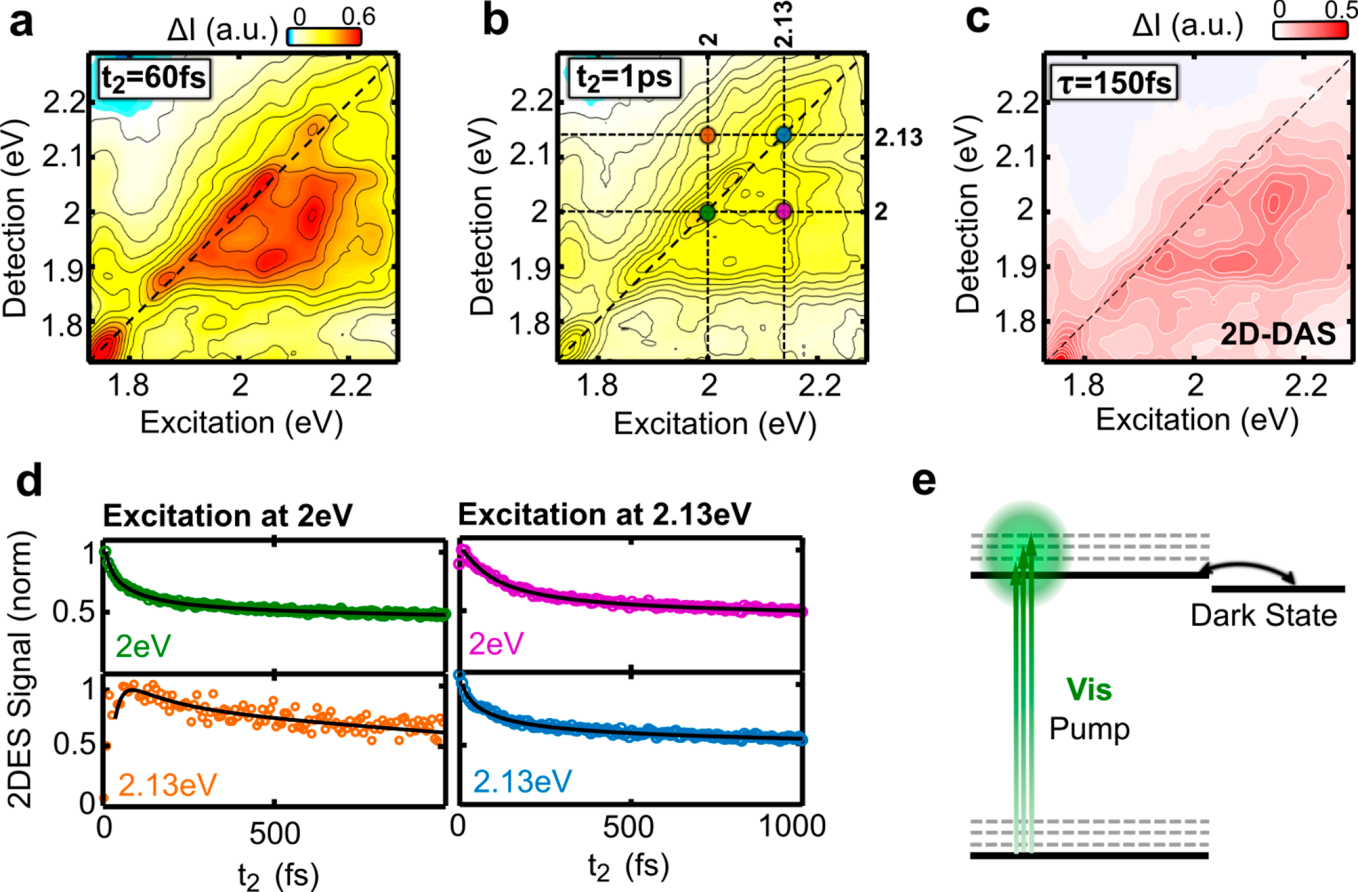
2DES maps of GNR-AHM-3 at (a) *t*_2_ = 60 fs and (b) *t*_2_ = 1 ps. (c) 2D-DAS corresponding to the exponential decay component of 150 fs. (d) Kinetics at the selected coordinates of the 2DES maps indicated in panel b. (e) Scheme representing the bright–dark state equilibrium formed after 150 fs.

At first, it may seem that a 150 fs decay of the SE contradicts the ∼10% PL quantum yield of GNR-AHM-3.^30^ Nonlinear exciton annihilation^59,60^ is inconsistent with the excitation fluence dependent data shown in Figure S7, which reveal the onset of many-body kinetics between 40 and 88 μJ/cm^2^, while all 2DES measurements were performed at lower fluences. To reconcile these observations, we propose that the photoexcited bright transition relaxes on the ultrafast time scale to a dark excited state, which is close enough in energy to the bright state to form a thermodynamic equilibrium with it, so that PL proceeds by thermal activation from this dark state, as depicted in Figure 4e. Although our spectroscopic techniques do not offer direct insight into the nature of the dark state, we speculate that, since the π-conjugated backbone is larger than the exciton size, electronic excitation relaxes away from the orbitals involved in the bright transition. Moreover, we note that the spectral shape of the steady-state emission closely follows that of absorption once spectral inhomogeneity is accounted for. That, along with the very small Stokes shift and the ∼6 ns PL lifetime (Figure S5), suggests that emission originates from the same transition responsible for absorption at the optical bandgap, and not from traps or different electronic states. Thus, a thermodynamic equilibrium among the dark and bright states accounts for all experimental observations. We note that a dark state has been reported for armchair GNRs before.^61^

In conclusion, 2DES reveals the excited state structure and dynamics of structurally defined GNRs with bulky AHM side groups, which suppress aggregation due to π–π stacking interactions, allowing one to study the photophysics of the isolated nanostructure. Our data indicate that the broad and featureless absorption spectrum of GNR-AHMs corresponds to an inhomogeneously broadened excitonic transition with strong vibronic coupling to the characteristic D, G, 2D, D+G, and 2G Raman bands. These results establish the importance of the local environment and vibronic coupling for understanding the optical properties of GNRs. In addition, we observe an ultrafast decay, on a 150–200 fs time scale, of the SE signal, which is consistent with the ultrafast relaxation of the excited state population from a bright to a dark state in thermal equilibrium with the emitting state. Taken together, our data suggest that the excited state dynamics of the GNRs is more similar to that of a large solvated organic molecule than to that of a quantum confined solid.

## Supporting Information Available

Synthesis details, EEMs of C78 and GNR-AHM-1, scheme of energy levels of GNRs, laser pulse spectra, TA spectrum for narrowband excitation, photoluminescence lifetime, and Raman spectrum and coherent oscillations in broadband TA data (PDF)

## Abbreviations

GNR: graphene nanoribbon
SWNT: single-walled nanotube
AHM: *N*-*n*-hexadecyl maleimide
EEM: excitation–emission map
TA: transient absorption
2DES: two-dimensional electronic spectroscopy
GSB: ground state bleaching
SE: stimulated emission
