# Supplementary material for: Electronic Structure of Isolated Graphene Nanoribbons in Solution Revealed by Two-Dimensional Electronic Spectroscopy
Source: Nano Lett. 2024 Jan 8;24(3):797–804. doi: 10.1021/acs.nanolett.3c02665 (PMC10811683; doi:10.1021/acs.nanolett.3c02665)
Supplement: Supplementary file 1 — nl3c02665_si_001.pdf [file nl3c02665_si_001.pdf]

Supporting Information

Electronic Structure of Isolated Graphene Nanoribbons in Solution Revealed by Two-Dimensional Electronic Spectroscopy

Tetsuhiko Nagahara[1,2§], Franco V. A. Camargo[3§], Fugui Xu[4*], Lucia Ganzer[1], Mattia Russo[1], Pengfei Zhang[4], Antonio Perri[1,†], Gabriel de la Cruz Valbuena[1], Ismael A. Heisler[5], Cosimo D’Andrea[1], Dario Polli[1], Klaus Müllen[6], Xinliang Feng[7], Yiyong Mai[4], and Giulio Cerullo[1,3*]

[1]Politecnico di Milano, Dipartimento di Fisica, Piazza L. da Vinci 32, 20133 Milano, Italy; [2] Department of Chemistry and Materials Technology, Kyoto Institute of Technology, 606-8585 Kyoto, Japan; [3]IFN-CNR, Piazza L. da Vinci 32, 20133 Milano, Italy; [4]School of Chemistry and Chemical Engineering, Frontiers Science Center for Transformative Molecules, Shanghai Jiao Tong University, 800 Dongchuan RD, Shanghai 200240, China; [5]Departamento de Física, Universidade Federal do Paraná, Caixa Postal 19044, 81531-990 Curitiba, Paraná, Brazil; [6]Max Planck Institute for Polymer Research, Ackermannweg 10, 55128, Mainz, Germany; [7]Department of Chemistry and Food Chemistry, Technische Universität Dresden, Mommsenstrasse 4, 01062 Dresden, Germany.

[†]Present address: NIREOS S.R.L., Via G. Durando 39, 20158 Milano, Italy

Materials: The GNR-AHM-1 and GNR-AHM-3 were synthesized according to the procedure described in our previous report.[1] The chemical synthesis of the GNR-AHM is illustrated in Scheme S1. Typically, a tetra-anthracenyl and dichloro-substituted oligo-phenylene 2 was synthesized by the Suzuki reaction of tetra-bromo- and dichloro-substituted oligophenylene 1 and 9-anthraceneboronic acid. Then, the monomer 3 with four AHM groups was synthesized by a Diels-Alder cycloaddition of compound 2 and *N*-n-hexadecyl maleimide. The desired PPP-AHM-1&3 were obtained via an AA-type Yamamoto polymerization of monomer 3. Finally, The PPP-AHM-1&3 were converted into arm-chair edged GNR-AHM-1&3 with uniform width (~1.7 nm) by employing the Fe(III) chloride-based Scholl reaction. The lengths and length dispersities of the GNRs were determined by their precursors, namely polyphenylenes before their cyclodehydrogenation, using gel permeation chromatography (GPC) measurements. The structural parameters of the PPP-AHM-1&3 were provided in Table S1.

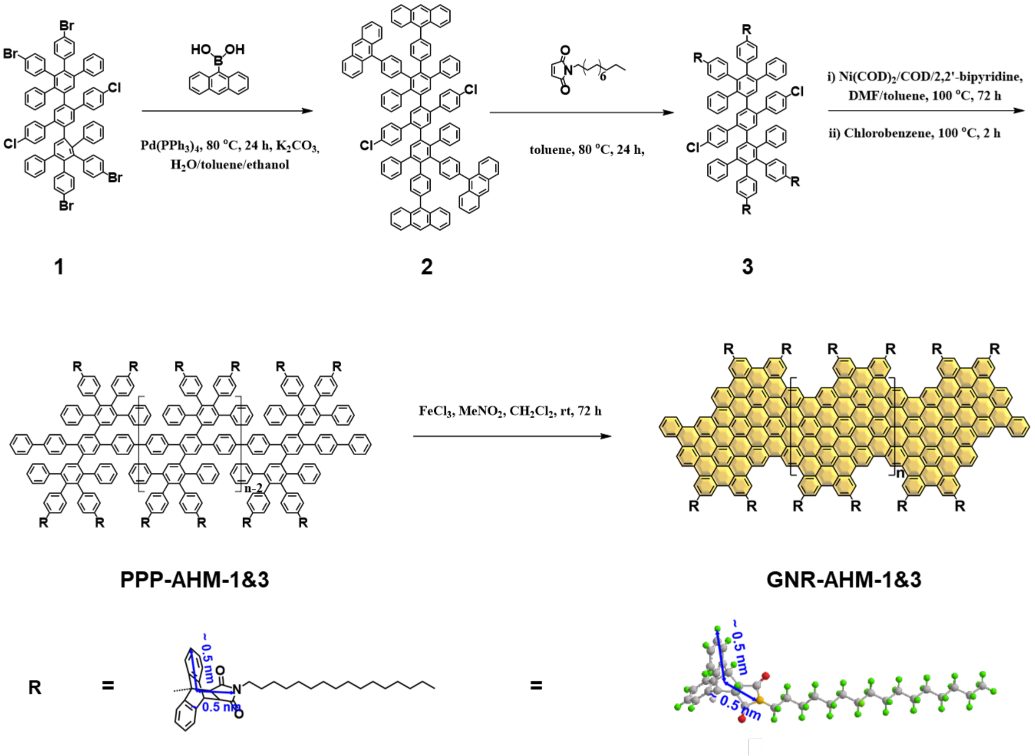

Scheme S1. The synthesis of GNR-AHM-1&3.

Table S1. The structural parameters of the PPP-AHM-1&3 and the length of obtained GNR-AHM.

Sample name	M_n (g/mol)	*Đ*	Degree of polymerization	Length of GNR (nm)
PPP-AHM-1	13500	1.42	4	6
PPP-AHM-3	124000	1.53	42	60

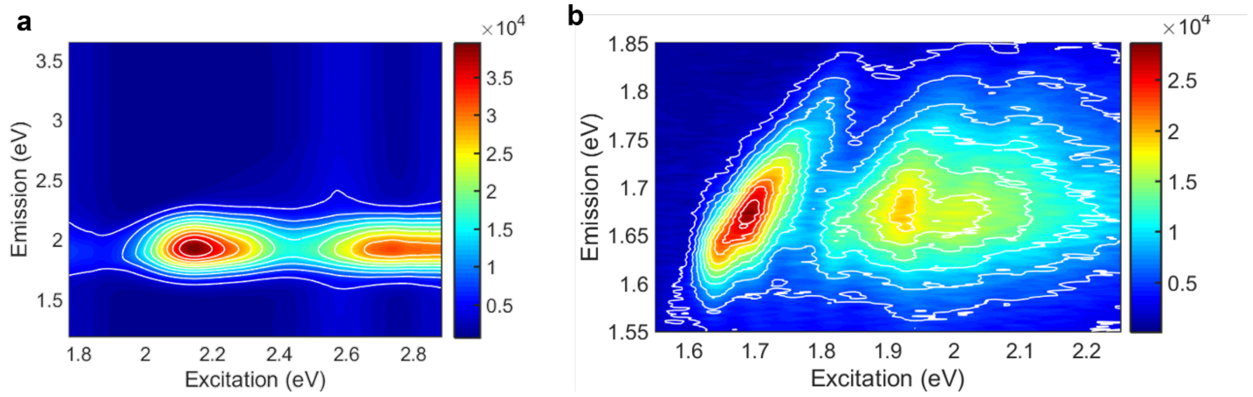

Figure S1. EEM of C78 (a) and of GNR-AHM1 (b)

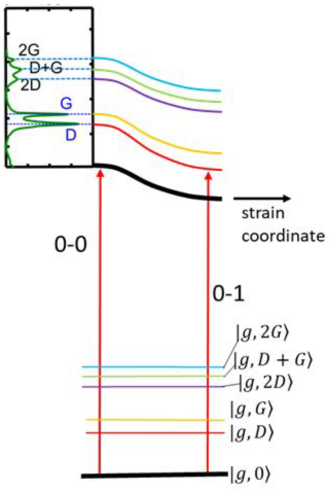

Figure S2. Scheme explaining the emission from the vibronic transitions of differently strained conformers in the EEM.

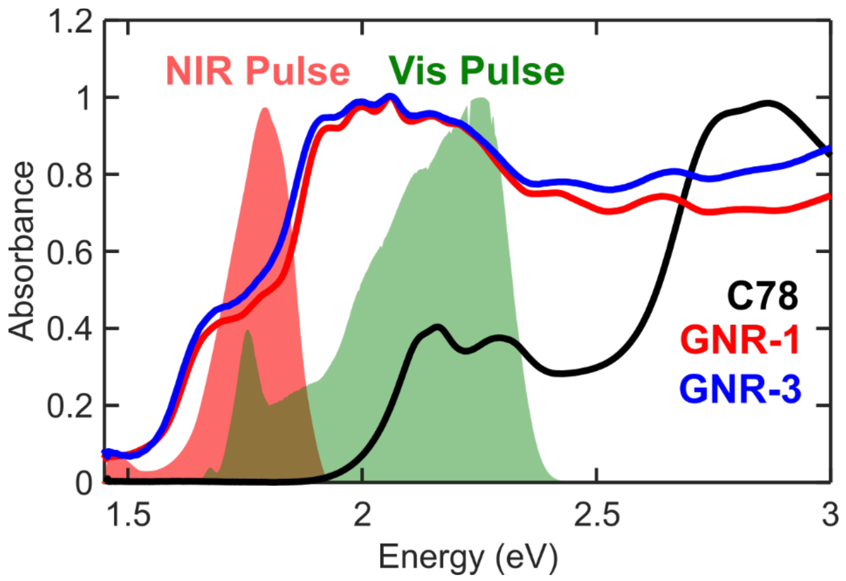

Figure S3. Spectra of pulses with absorption spectrum of the GNRs.

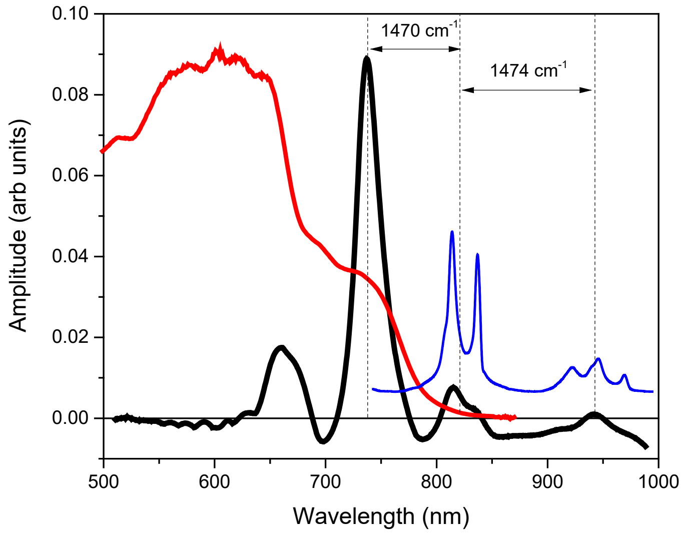

Figure S4. Red curve – linear absorption; black line – TA at 0 fs; blue line – Raman spectrum.

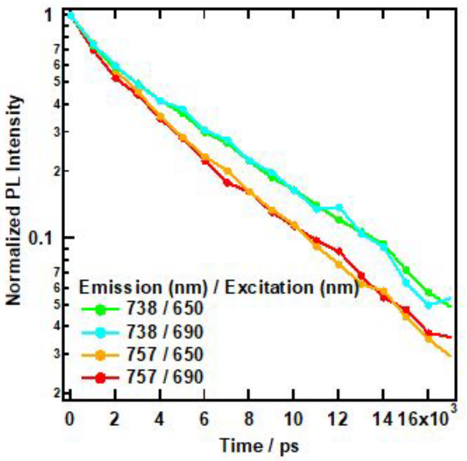

Figure S5. Time resolved photoluminescence dynamics of the GNRs for different excitation and emission wavelengths. The emission at 757 nm has a lifetime of 5.4 ns, shorter than that at 690 nm, which is 6.1 ns.

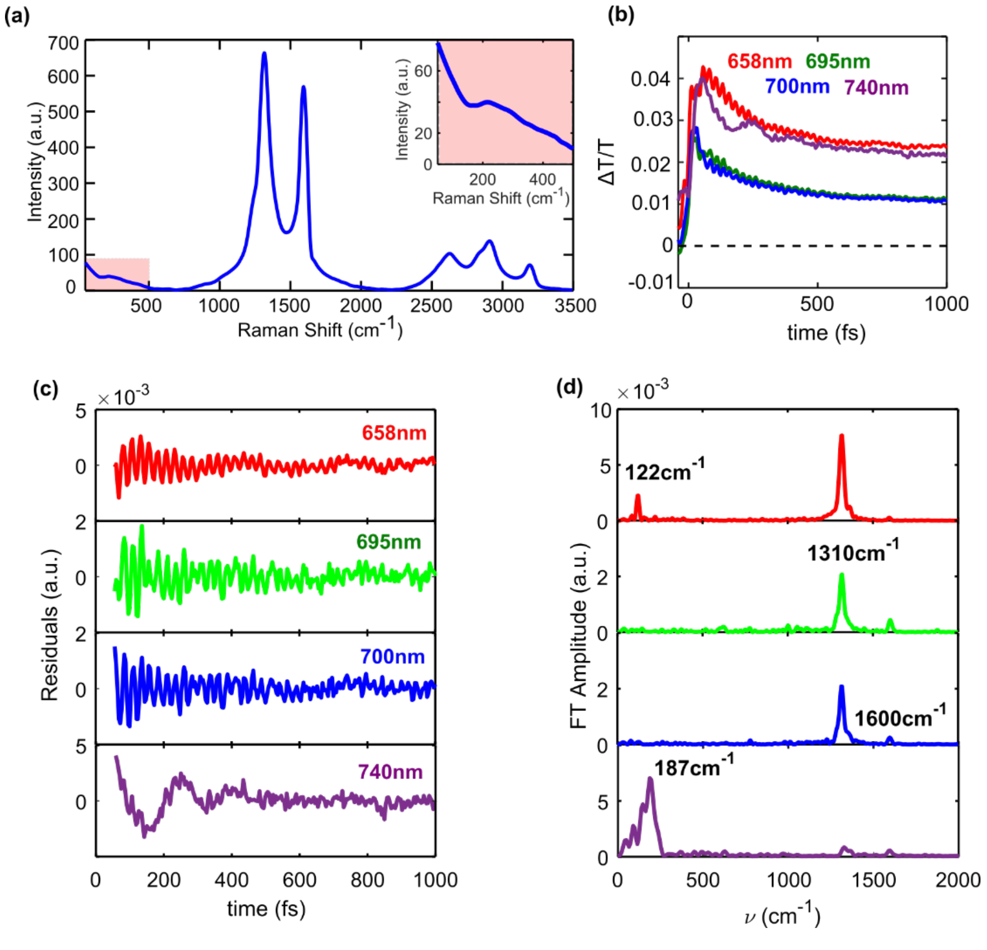

Figure S6. (a) Raman spectrum of GNR-AHM-3 in powder. (b) Time traces of transient absorption signal at different probe wavelengths, (c) their oscillatory residuals after subtracting kinetic terms and (d) the power spectra obtained by fast Fourier transformation (FFT). Raman peaks in D/G and low frequency regions are clearly seen.

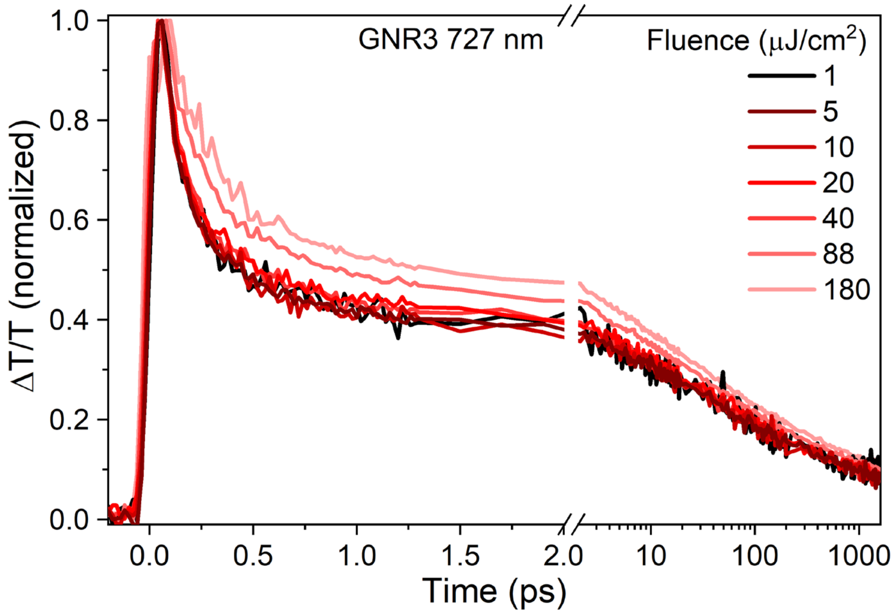

Figure S7. Normalized transient absorption kinetics of GNR-AHM-3 in toluene at 727 nm (1.705 eV) probe wavelength following excitation at 730 nm. The time axis is linear up to 2 ps and logarithmic beyond that. The onset of many-body kinetics lies between 40 and 88 μJ/cm^2.

References

1. Yinjuan Huang, Fugui Xu, Lucia Ganzer, Franco V. A. Camargo, Tetsuhiko Nagahara, Joan Teyssandier, Hans Van Gorp, Kristoffer Basse, Lasse Arnt Straasø, Vaiva Nagyte, Cinzia Casiraghi, Michael Ryan Hansen, Steven De Feyter, Deyue Yan, Klaus Müllen, Xinliang Feng, Giulio Cerullo, Yiyong Mai, Intrinsic Properties of Single Graphene Nanoribbons in Solution: Synthetic and Spectroscopic Studies. *J. Am. Chem. Soc*. 2018, *140*, 10416–10420.
